# Reference-free clustering as an epidemiological tool for Mycobacterium tuberculosis lineage typing

**DOI:** 10.1099/mgen.0.001759

**Published:** 2026-06-24

**Authors:** Aureliana F. C. Chilengue, Daniel J. Whiley, Kate Cox, Maria Rosa Domingo-Sananes, Conor J. Meehan

**Affiliations:** 1 Department of Biosciences, Nottingham Trent University, Nottingham, UK; 2 Department of Physiological Sciences, Faculty of Medicine, Eduardo Mondlane University, Maputo, Mozambique; 3 Medical Technologies Innovation Facility, Nottingham Trent University, Nottingham, UK; 4 Quadram Institute Bioscience, Norwich, UK; 5 University of East Anglia, Norwich, UK; 6 Unit of Mycobacteriology, Institute of Tropical Medicine, Antwerp, Belgium

**Keywords:** lineage assignment, *Mycobacterium tuberculosis*, reference-free tools, strain typing, whole-genome sequencing

## Abstract

Whole-genome sequencing (WGS) of *Mycobacterium tuberculosis* (Mtb) is widely used in epidemiological investigations of recent transmission events, resulting in high-resolution strain typing. Accurate and rapid strain typing is essential for informing outbreak investigations and guiding tuberculosis control strategies. However, the gold-standard reference-guided SNP-calling pipeline currently used for strain typing relies on computationally intensive reference-mapping approaches, making it challenging to perform in many high-burden, resource-limited settings, where simplified and scalable genomic tools are urgently needed. To address these limitations, we explored reference-free methods for medium-resolution epidemiology, namely, Mtb strain (lineage) typing, using a dataset of 535 complete genomes spanning the human- and animal-adapted lineages. Illumina paired-end reads were simulated from each complete genome, assembled and analysed using three reference-free, *k*-mer-based tools: MASH, PopPUNK and SKA2 (split *k*-mer analysis). Genetic distances were generated for each method and compared with a ground truth lineage assignment from TB-Profiler. Our results demonstrated that reference-free methods can effectively distinguish Mtb lineages, with SKA2 showing the most promising performance across all datasets. SKA2 consistently recovered lineage and sub-lineage structure with high accuracy, demonstrating strong potential as an alternative to traditional WGS workflows. These findings highlight the utility of reference-free methods, particularly SKA2, for enabling accessible, scalable and rapid Mtb strain typing, while supporting genomic epidemiology with low computational resources.

**Impact Statement**

Tuberculosis (TB) remains one of the leading causes of mortality from infectious diseases worldwide. Over the past decade, whole-genome sequencing (WGS) has been increasingly used to support surveillance and outbreak investigations by enabling high-resolution strain typing to inform disease control efforts. However, the gold-standard reference-guided SNP-calling pipelines used for WGS-based strain typing are computationally intensive, limiting their routine use in many resource-limited, high-burden settings where rapid and scalable strain typing is essential for effective TB control. In this study, we evaluate reference-free tools for *Mycobacterium tuberculosis* strain typing using a large and diverse collection of complete genomes and simulated sequencing reads for each complete genome. We show that reference-free tools can reliably recover lineage structure, with approaches such as split *k*-mer analysis performing efficiently across datasets while remaining computationally efficient and easy to deploy. This work highlights the potential of reference-free methods to enable accessible, scalable and rapid strain typing, supporting broader adoption of genomic epidemiology in low-resource settings.

## Data Summary

All complete *Mycobacterium tuberculosis* genomes analysed in this study were retrieved from the NCBI RefSeq and GenBank databases, including genomes previously reported by Behruznia *et al*. [1] and additional complete genomes available up to April 2025. Simulated Illumina paired-end reads were generated from these genomes using InSilicoSeq v1.5.4. Accession numbers for all genomes included in the analysis are provided in Table S1.

## Background

*Mycobacterium tuberculosis* (Mtb), the causative agent of tuberculosis (TB), is the leading cause of mortality globally due to pulmonary infection (WHO, 2023). According to the WHO (2023) [2], Mtb infects nearly 100 million people annually, resulting in over 10.8 million active cases and 1.25 million deaths worldwide. The burden is disproportionately high in South-East Asia and Sub-Saharan Africa, where most cases are reported.

Mtb comprises ten human-adapted lineages and several animal-adapted lineages, including La1-La3, *Mycobacterium microti* and *Mycobacterium pinnipedii* [3]. Each of these lineages exhibits unique epidemiological and phylogeographical characteristics [3], which are critical for understanding the global distribution and impact of TB. Lineages 2 and 4 are the most widespread, while lineage 1 causes the most infections in absolute numbers. Conversely, some lineages, such as 5–10, are more geographically restricted and often underrepresented in public datasets [1]. Assigning lineage designations (often referred to as strain typing) to clinical strains is becoming increasingly important as evidence arises linking specific lineages to increased virulence and decreased drug susceptibilities [4].

Recently, public health surveillance has been increasingly supported by molecular techniques to better understand the epidemiology of Mtb. The current gold standard for Mtb molecular epidemiology is whole-genome sequencing (WGS), which enables the identification of transmission clusters by detecting genetic variants such as SNPs [5]. This approach also allows strain typing of human- and animal-adapted lineages, as well as their hierarchy of sub-lineages, through the analysis of variant calls and lineage-defining SNPs [5]. After SNPs are identified, strain typing is typically performed by comparing isolate-specific SNP patterns against a curated set of phylogenetically informative markers that reflect shared ancestry [6]. These lineage-defining SNPs are used to classify isolates into hierarchical groups through phylogenetic reconstruction or barcode-based schemes, providing a medium-resolution and reproducible framework for characterizing population structure and tracking evolutionary relationships [6,8].

However, despite its strengths, WGS analysis relies on complex, computationally intensive pipelines to call SNPs, demanding significant bioinformatics skills and large computing resources [9]. The gold standard for Mtb WGS is a mapping-based approach using the type strain H37Rv (a lineage 4 strain) as the reference. The dependence of the mapping-based approach on the H37Rv reference strain may introduce biases and limitations, as this reference does not adequately represent the global diversity of Mtb [10]. These requirements make WGS bioinformatics analysis challenging to perform in many endemic clinical settings [11], especially when there is a need to deliver rapid results through simplified, low-computational-demand workflows that do not require highly trained bioinformaticians.

These limitations can be particularly challenging given the fact that the rapid identification of Mtb lineages is crucial for predicting potential future outbreaks [8] and for evaluating the effectiveness of disease control measures, including therapeutics and vaccines [6], whose efficacy may vary depending on the strain type [12]. Although mapping-based SNP-calling methods are widely regarded as the most valid approach for defining phylogenetic groupings with high confidence [6,7, 13], the comparably low level of genomic diversity in Mtb strains makes it challenging to achieve high-resolution discrimination within the complex [7], particularly at finer phylogenetic scales [6]. These limitations highlight the need to complement mapping-based strategies with non-SNP-mapping methods, including reference-free approaches, to ensure scalable and consistent classification across diverse epidemiological contexts.

Reference-free methods have emerged as a promising alternative for epidemiology studies and have been widely used for strain typing and transmission analysis. These methods bypass the need for alignment or mapping to a set reference and undertake variant calling by directly comparing sequence content. As a result, they offer several advantages, including reduced computational complexity and processing time, and minimized reference bias, resulting in greater flexibility when analysing diverse strains [14,15].

Such approaches have the potential to capture a more complete picture of genetic diversity, including in genomic regions that might be missed by reference-based approaches [15]. Examples include *k*-mer-based methods such as MASH [16] and PopPUNK [14], which estimate genetic distances between isolates using shared *k*-mer content. Another tool, SKA2 (split *k*-mer analysis) [17], uses split *k*-mers to identify SNPs between genomes without the need for alignment. These tools have shown variable accuracy for Mtb in the past, ranging from low correlation for transmission detection [14] to similar results compared to the gold standard [17], albeit all on simulated data. This study aims to explore the accuracy of these reference-free tools for rapid and accurate strain typing of Mtb, specifically the identification of Mtb (sub-)lineages as a way to enable more efficient and accessible genomic epidemiology in several settings [18], particularly in low-resource settings where limited computing power, lack of dedicated server infrastructure and reduced access to specialist bioinformatics expertise constrain the use of conventional reference-based workflows.

## Methods

### Dataset

#### Selection and characterization of the genomes

A total of 535 complete and closed (single contig) genome assemblies from human-adapted (L1-L9) and animal-adapted (La1, La2, La3, *M. microti* and *M. pinnipedii*) lineages across Mtb were selected for this study (Table S1, available in the online Supplementary Material). This dataset consisted of those from a previously published study [1], which collected all closed genomes from NCBI up until 2023. The same procedure was repeated to complement this collection with all complete Mtb genomes from NCBI up until April 2025. Quality assessment of the assemblies was performed using BUSCO v5.4.7 [19] to ensure that only those with ≥95% completeness were included in the study.

Lineage assignment was done using TB-profiler version 5.2 [20] and was defined as the ground-truth assignments. Spoligotype patterns were also estimated for each isolate from its genome using the --spoligotype option in TB-Profiler version 5.2 [8].

#### Simulated reads

As raw sequencing reads were not available for all the genomes included in this study, paired-end Illumina-like reads (300 bp) were simulated from 535 complete Mtb genomes using InSilicoSeq v1.5.4 [21] to a depth of ~100X. The simulation employed the built-in MiSeq error model, which reproduces empirical sequencing features such as substitution, insertion and deletion errors, GC bias and quality score distributions. This model is derived from aligned reads in the PRJEB20178 dataset and accurately mimics Illumina MiSeq sequencing characteristics [21]. The simulated reads were output as compressed FASTQ files for each genome and used for further analysis.

#### Assembly of simulated reads

The simulated paired-end reads were assembled using SKESA v2.4.0 [22], a conservative *de novo* assembler optimized for Illumina sequencing data. For each genome, paired FASTQ files (*R1.fastq.gz and *R2.fastq.gz) were provided to SKESA using default parameters. The resulting assemblies were saved in FASTA format and used for downstream analyses.

### SNP distance estimations using assembly-based and read-mapping approaches

The 535 genome assemblies were compared pairwise using DNAdiff v1.3 from the MUMmer4 tool suite [23]. The resulting GSNP counts, where each SNP is bound by 20 exact matches to reduce false positives, were then used to compute pairwise SNP distances between isolates. DNAdiff was included as an assembly-based, reference-free comparator, evaluated alongside *k*-mer-based approaches for consistency with TB-Profiler lineage assignments.

To provide a representative reference-based comparator, short-read SNP distances were additionally inferred using MTBseq [24]. As raw sequencing reads were not available for all isolates, simulated short reads were processed using the standard MTBseq pipeline, including mapping to the H37Rv reference genome and variant calling under default quality filters. Pairwise SNP distances derived from MTBseq were evaluated alongside DNAdiff and the reference-free methods, enabling direct comparison of reference-free distance estimates against both assembly-based and short-read mapping–based approaches with respect to TB-Profiler lineage assignments.

### Reference-free methods for clustering genomes

Three reference-free methods were employed in our analysis: PopPUNK [14], MASH [16] and SKA2 [17]. These methods were applied to complete genomes, simulated reads and assembled genomes to assess their consistency across different data types. Unless otherwise stated, all tools were run using their default parameters.

We ran PopPUNK version 2.6.0 [14] using the ***poppunk --create-db*** command and evaluated multiple *k*-mer ranges, including the default (13–29), an intermediate (21–41) and an extended (29–61) range. In addition, the sketch size was increased from the default value (10,000) to 100,000 to assess the impact of sketch resolution on distance estimation.

Based on this assessment, an extended *k*-mer range of 29–61 and a sketch size of 100,000 were selected and used for all downstream analyses. Pairwise core and accessory genome distances were estimated using MinHash sketches based on shared *k*-mer content and used to infer population structure across the dataset. For downstream clustering and comparative analyses, only core genome distances were used, as it better captures conserved genomic variation relevant for lineage and strain differentiation. Core distance matrices were subsequently extracted using the ***poppunk_extract_distances.py*** command .

Since MASH [16] operates using a single *k*-mer size, we ran the ***mash sketch*** (version 2.3) command using the default parameter settings (*k*=21, sketch size=1,000), as well as configurations using a larger *k*-mer size (k=31) and increased sketch sizes up to 10,000. A *k*-mer size of 31 and a sketch size of 10,000 were used for all downstream analyses. These sketches were then compared using the MinHash algorithm as above. The resulting pairwise distances were extracted using the ***mash dist*** command and used to cluster the genomes according to their genetic similarities.

For SKA2 [17] version 0.4.0, we used the ***ska build*** command to generate the split *k*-mers with a variable middle base. We evaluated multiple *k*-mer sizes, including the default (*k*=17), as well as *k*=21, 31 and 41, to assess their impact on distance estimation. A *k*-mer size of 31 was selected for all downstream analyses. These split *k*-mers were used as keys in a hash map, with their middle bases stored as values. After building the dictionaries for all genomes, the genetic distances between all pairs of genomes were calculated using the ***ska distance*** command by iterating over every pair of samples to compare their split *k*-mer dictionaries. For each shared split *k*-mer, SKA2 assessed whether the middle nucleotide matched between genomes by comparing the nucleotide stated at that position.

### Genetic distance distribution

To compare the genetic distance distributions within and between lineage groupings, we used the distance matrices generated by each method (DNAdiff, MTBseq, PopPUNK, MASH and SKA2) and combined them with the TB-Profiler-defined lineage assignments. Using a custom R script, we matched each genome pair to its corresponding lineage classification, labelling pairs as ‘within-lineage’ or ‘between-lineage’, and assigned them to the appropriate lineage group or the ‘between’ category. We then visualized these distance distributions using histograms and density plots with the ggplot2 package [25], applying custom colour schemes to highlight lineage-specific patterns. This approach enabled us to evaluate how each method addressed genetic distances and whether it effectively maintained separation between distinct phylogenetic groups.

### Reference-free clustering and lineage delineation

Using the genetic distance matrices generated from the four methods, we constructed distance-based neighbour-joining (NJ) trees using the NJ function implemented in the APE package (version 5.7.1) [26] in R (version 4.3.2). NJ was chosen to provide a distance-based representation of genetic similarity without imposing hierarchical or linkage-based clustering assumptions, allowing consistent comparison of grouping patterns across different distance estimation approaches.

These trees were then visualized using Interactive Tree of Life [27] to illustrate the separation and delineation of lineages for each method.

### Cluster membership accuracy

A lineage cluster membership accuracy ratio was used to evaluate how accurately each method categorized the isolates into clusters corresponding to the TB-Profiler lineage designations. Since lineages are monophyletic groups, we used a tree-based phylogenetic clustering approach to determine if lineages formed clades in the distance-based NJ trees constructed by each of the SNP distance estimation approaches. This was conducted to assess the ability of each approach to accurately resolve a given lineage-based cluster.

For this analysis, we used a custom Python script that took a tree file in Newick format and a file containing all the isolate names of a given lineage. This methodology is illustrated in Fig. S1. For a given lineage-based cluster (as determined by TB-Profiler), the most recent common ancestor (MRCA) node was determined within the tree, and the subtree that contained all the taxa that descended from that MRCA was extracted from the parent tree. The number of taxa in the original lineage cluster and the number of taxa in the extracted subtree were calculated. Subsequently, the total count of isolates in each lineage was divided by the subtree count from each method. If these two sets were identical (i.e. the subtree of the cluster contained only taxa that were designated to be in that lineage-based cluster), the method accurately grouped that lineage, giving a ratio of 1. A ratio below 1 reflected either misclassification, where isolates assigned to one sub-lineage were grouped into a different sub-lineage, or extended clustering, where isolates without sub-lineage designation were grouped into a specific sub-lineage cluster.

To identify the isolates being grouped incorrectly by each method (i.e. a clustering ratio <1), we coloured the subtree based on lineage assignment (Fig. S1). This allowed for visual inspection of isolate clustering and identification of the cause of over-clustering. Spoligotype patterns were then used to further explain any unexpected grouping of these genomes.

Genomes lacking sub-lineage designations were only labelled at the higher lineage level. Therefore, we evaluated clustering based on the available designations and did not assess separation at finer levels where assignments were missing.

### Code availability

All scripts used for this analysis are available in the public GitHub repository. The version used to generate the results reported in this manuscript corresponds to git tag v1.0.0. The analysis code has been archived on Zenodo and is available under the DOI 10.5281/zenodo.20020152, corresponding to the exact version used to generate the results reported in this manuscript.

### Data availability

The genome assemblies used in this study have been archived on Zenodo and are available under the DOI 10.5281/zenodo.20021113, enabling access to the complete set of sequences used in the analyses.

## Results

Our dataset comprised 535 closed Mtb genomes covering human-adapted and animal-associated lineages. Lineage assignment was performed using TB-Profiler, which served as the ground truth for reference-free analyses. Pairwise whole-genome SNP distances were computed using DNAdiff for genome-to-genome comparisons, and short-read SNP distances were additionally inferred using MTBseq. Alongside this, three *k*-mer-based, reference-free methods (PopPUNK, MASH and SKA2) were systematically analysed in concordance with TB-Profiler lineage assignments using the complete genomes, simulated reads or re-assembled genomes as input.

### Selection of *k*-mer and sketch parameters

To determine appropriate *k*-mer sizes and sketch sizes for reference-free tools, we evaluated their impact based on distance correlations with a benchmarking method, clustering stability and consistency with established Mtb lineage assignments.

Comparison of different *k*-mer ranges in PopPUNK showed that lineage-level clustering was largely unaffected across parameterizations. However, the use of an extended *k*-mer range (29–61), combined with an increased sketch size of 100,000, resulted in improved resolution at the sub-lineage level, particularly in terms of grouping genomes according to lineage assignments (Fig. S2).

A similar pattern was observed for MASH. Increasing the *k*-mer size from the default (*k*=21) to *k*=31 and enlarging the sketch size to 10,000 improved lineage- and sub-lineage-level grouping relative to the default configuration, while preserving consistent overall clustering patterns (Fig. S3).

Increasing the split *k*-mer size from the default (*k*=17) to *k*=31 in SKA2 resulted in improved resolution of lineages and sub-lineages. Further increases (*k*=41 and *k*=51) did not lead to additional changes in SNP distance distributions or lineage grouping. Consistent grouping of genomes according to lineage was observed from *k*=31 onwards (Fig. S4).

Based on these results, an extended *k*-mer range of 29–61 with a sketch size of 100,000 was selected for PopPUNK, a *k*-mer size of 31 with a sketch size of 10,000 was selected for MASH, and a split *k*-mer size of 31 was selected for SKA2.

### Genetic distance distributions across Mtb lineages using reference-free approaches

**Fig. 1.**
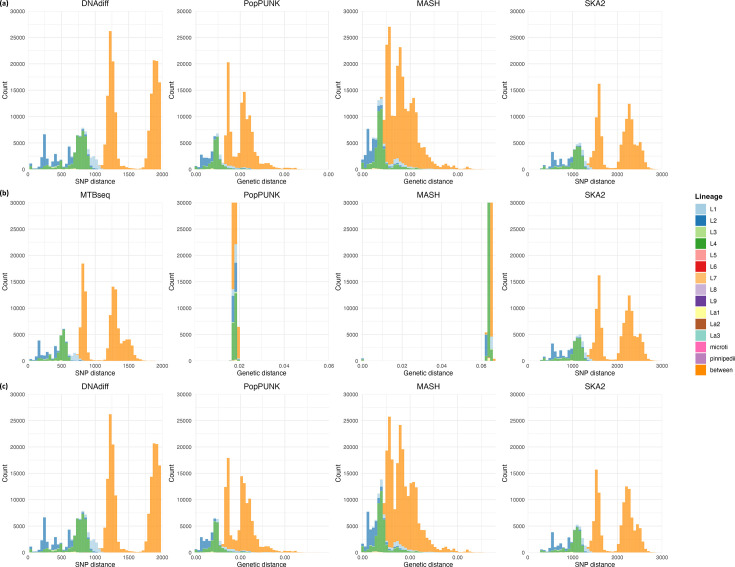
Comparative distributions of genetic distances within and between Mtb lineages using complete genomes (**a**), simulated reads (**b**) and assemblies generated from simulated reads (**c**). SNP-based methods (DNAdiff, MTBseq and SKA2) report pairwise SNP distance distribution, while *k*-mer-based methods (PopPUNK and MASH) report genetic distance distribution derived from *k*-mer similarity. DNAdiff was applied to complete genomes and SKESA assemblies (**a, c**) as an assembly-based benchmark tool, while MTBseq was applied only to simulated reads (**b**) as a reference-based short-read comparator. Inter-lineage comparisons are shown in orange, while intra-lineage comparisons are coloured by lineage.

We used the genetic distance distributions within and between lineages based on the various methods and datasets to assess their robustness at differentiating lineages from each other. For the complete genomes, DNAdiff and SKA2 SNP-based distances produced the strongest separation between intra-lineage and inter-lineage distances (Fig. 1). In contrast, PopPUNK and MASH *k*-mer-based distances showed broader intra-lineage distributions and greater overlap with inter-lineage distributions of genetic distances, with MASH exhibiting the most pronounced overlap. Stratification of genomes based on sequencing platform (Table S1) did not impact these findings for any tool analysed.

When applied to simulated short reads, SKA2 produced well-separated intra-lineage and inter-lineage distance distributions, consistent with those obtained using the read-mapping SNP pipeline MTBseq (Fig. 1b). In contrast, PopPUNK and MASH showed substantial overlap between intra-lineage and inter-lineage distance distributions when run directly on reads (Fig. 1b).

After assembling the simulated reads, PopPUNK and MASH regained the expected separation, and SKA2 showed consistent distance patterns across all data types (Fig. 1c).

### Reference-free tools for delineating the lineages of Mtb

All three reference-free methods (PopPUNK, MASH and SKA2) recovered the same major grouping structure as the TB-Profiler lineage classifications when given complete genomes as input (lineages 1–9 and animal-associated lineages La1, La2, La3, *M. microti* and *M. pinnipedii*). The resulting clustering trees matched the expected grouping patterns based on the full-genome DNAdiff distance-based clustering (Fig. S5). A visual comparison of the resulting trees revealed broadly similar topologies across the methods. Minor differences in branch resolution and cluster compactness were observed for PopPUNK and MASH, whereas SKA2 showed clear separation between lineage groupings. All methods produced stable and coherent grouping behaviour when run on the complete genomes.

Using the raw simulated reads as input, SKA2 recovered the same major lineage-level grouping structure as the TB-Profiler lineage classifications, and the resulting clustering trees were consistent with the grouping patterns derived from the reference-based read-mapping MTBseq. In contrast, PopPUNK and MASH did not consistently recover several lineage groupings when applied directly to reads (Fig. S6). After assembling the simulated reads, PopPUNK and MASH produced lineage groupings similar to those observed with the complete genomes, and SKA2 consistently showed strong lineage-level grouping across all data types (Fig. S7).

### Reference-free tools reveal varying performance in clustering Mtb genomes across lineage levels

For the complete genomes included in this study, all three reference-free methods achieved perfect classification at the lineage level, with a cluster membership ratio of 1.0 (Fig. S8) across all major Mtb lineages, demonstrating alignment with DNAdiff and correctly grouping genomes according to TB-Profiler lineage assignment.

At the sub-lineage level, some notable deviations were observed in groupings. Approximately 62% of the genomes broadly assigned to lineage 3 and lacking sub-lineage designation were grouped into sub-lineage L3.1 by all three reference-free methods and DNAdiff, with a consistent drop in the ratio below 1.0 (Fig. S9). Spoligotype analysis showed that both the L3 and L3.1 genomes in this cluster shared the CAS1 pattern.

Similarly, 90.6% of isolates from sub-lineages L4.8, L4.9 and L4.7 were grouped within L4.6 by PopPUNK and MASH, and all these genomes displayed LAM spoligotype patterns. Additionally, 13.3% of La1.7 isolates clustered with La1.8, consistent with the shared BOV-family spoligotype pattern (Fig. S9).

The clustering performance at finer lineage resolutions (e.g. sub-sub-lineage) was generally accurate for SKA2 and consistent with DNAdiff and TB-Profiler. However, 85.7% of genomes originally assigned to lineage 3 were grouped within the sub-sub-lineage L3.1.2, across all methods, including SKA2 and DNAdiff (Fig. S10). A similar pattern was seen for L3.1.1, where 85.7% of lineage 3 genomes were clustered into this group by PopPUNK and MASH. These two methods also grouped genomes originally assigned to L1.1 into L1.1.3, while 37.5% of genomes from La1.8.1 were clustered with La1.7. At the sub-sub-sub-lineage level, all methods correctly group the genomes with a lineage designation (ratio=1.0) according to their lineages (Fig. S11), although only 29% (157/535) of genomes had a sub-sub-lineage designation by TB-Profiler.

**Fig. 2.**
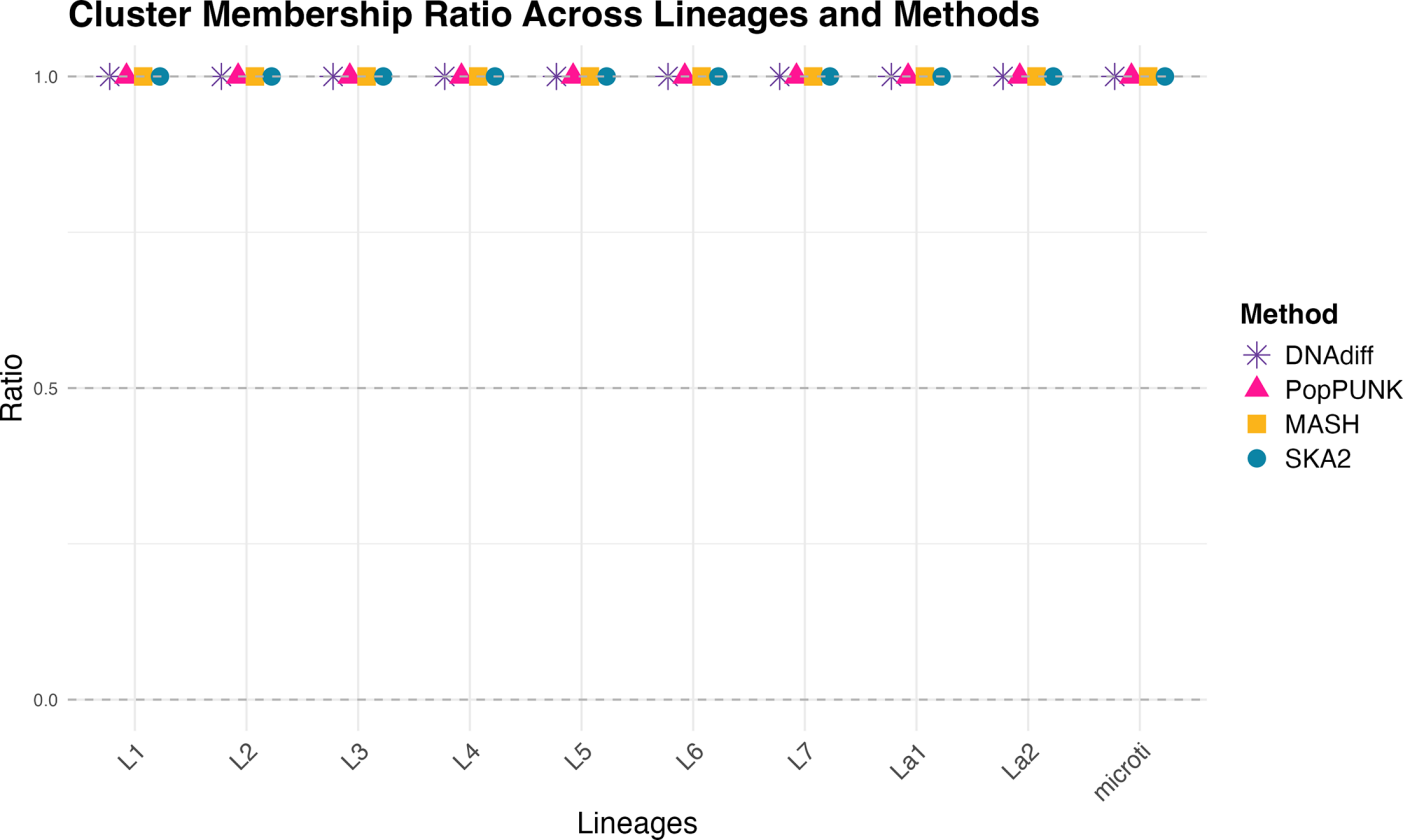
Lineage cluster membership accuracy ratio for each method using assembled simulated reads. A ratio of 1.0 indicates perfect agreement between each method’s clustering results and the TB-Profiler–assigned lineage groups. Ratios below 1.0 reflect over-clustering, where genomes from different lineage groups were incorrectly merged into the same cluster. A total of 535 genomes were included in this analysis.

When applied to simulated reads, PopPUNK and MASH showed consistently poor clustering performance across all lineage levels, with cluster membership ratios falling below 1.0 for most lineages, particularly for PopPUNK (Figs S12–S15). At all lineage levels, genomes from different lineages were frequently grouped together, with inter-lineage mixing. At the lineage level, correct clustering was observed only for L7, which included two genomes, for both PopPUNK and MASH. At finer lineage resolutions, correct clustering by MASH was observed only in a small number of cases involving very limited group sizes, including sub-sub-sub-lineages L1.2.1.1, L4.1.1.1 and L4.2.2.2 (each comprising two genomes) (Fig. S15), whereas PopPUNK did not cluster correctly at these finer resolutions. Following genome assembly, clustering performance for both PopPUNK and MASH improved, with results matching those obtained from complete genome sequences (Figs2^5^). In contrast, SKA2 produced identical results for simulated reads, assembled genomes and complete genomes.

**Fig. 3.**
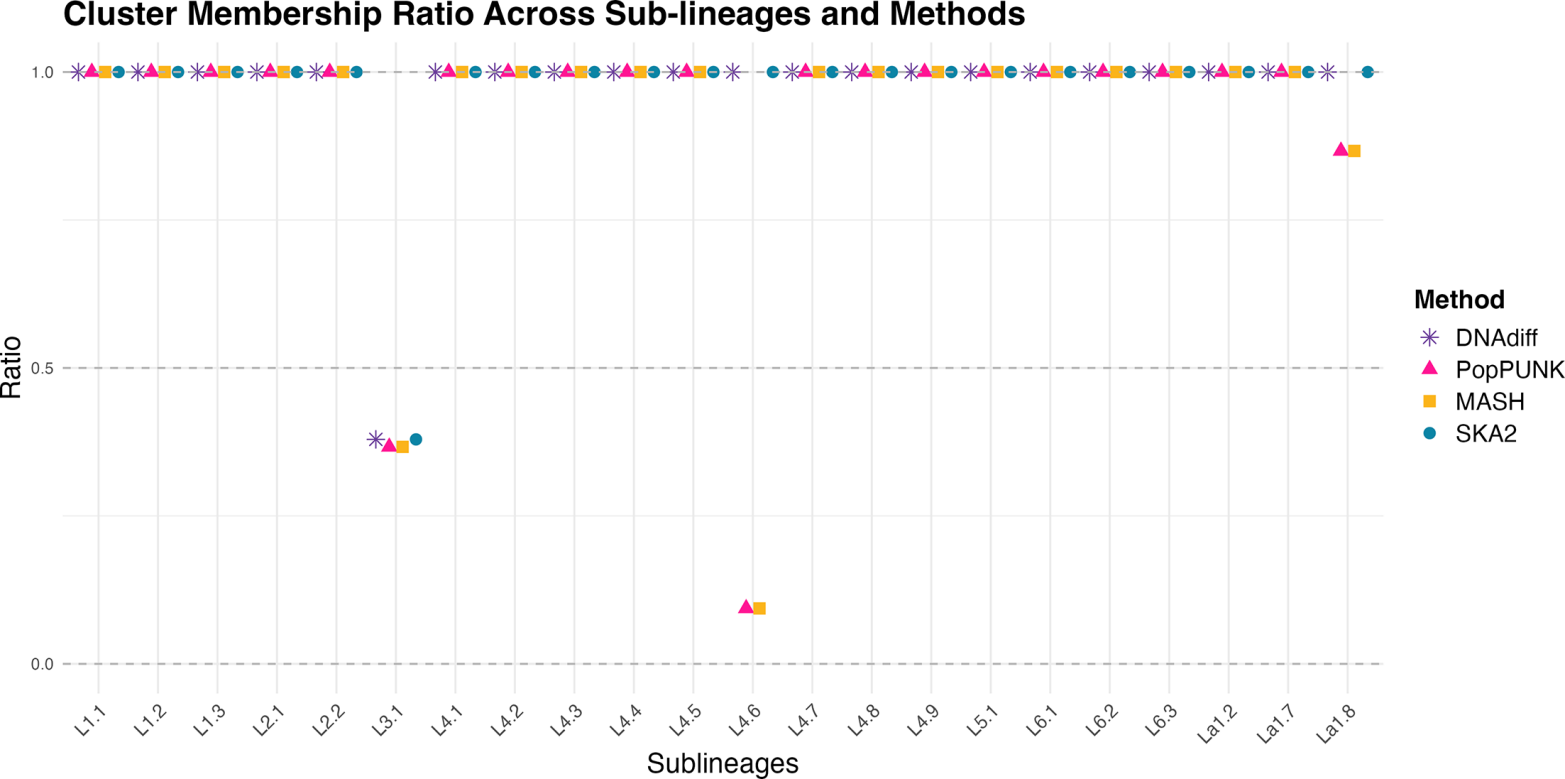
Sub-lineage cluster membership accuracy ratio for each method using assembled simulated reads. A ratio of 1.0 indicates perfect agreement between each method’s clustering results and the TB-Profiler–assigned lineage groups. Ratios below 1.0 reflect over-clustering, where genomes from different sub-lineage groups were incorrectly merged into the same cluster. A total of 494 genomes were included in this analysis.

**Fig. 4.**
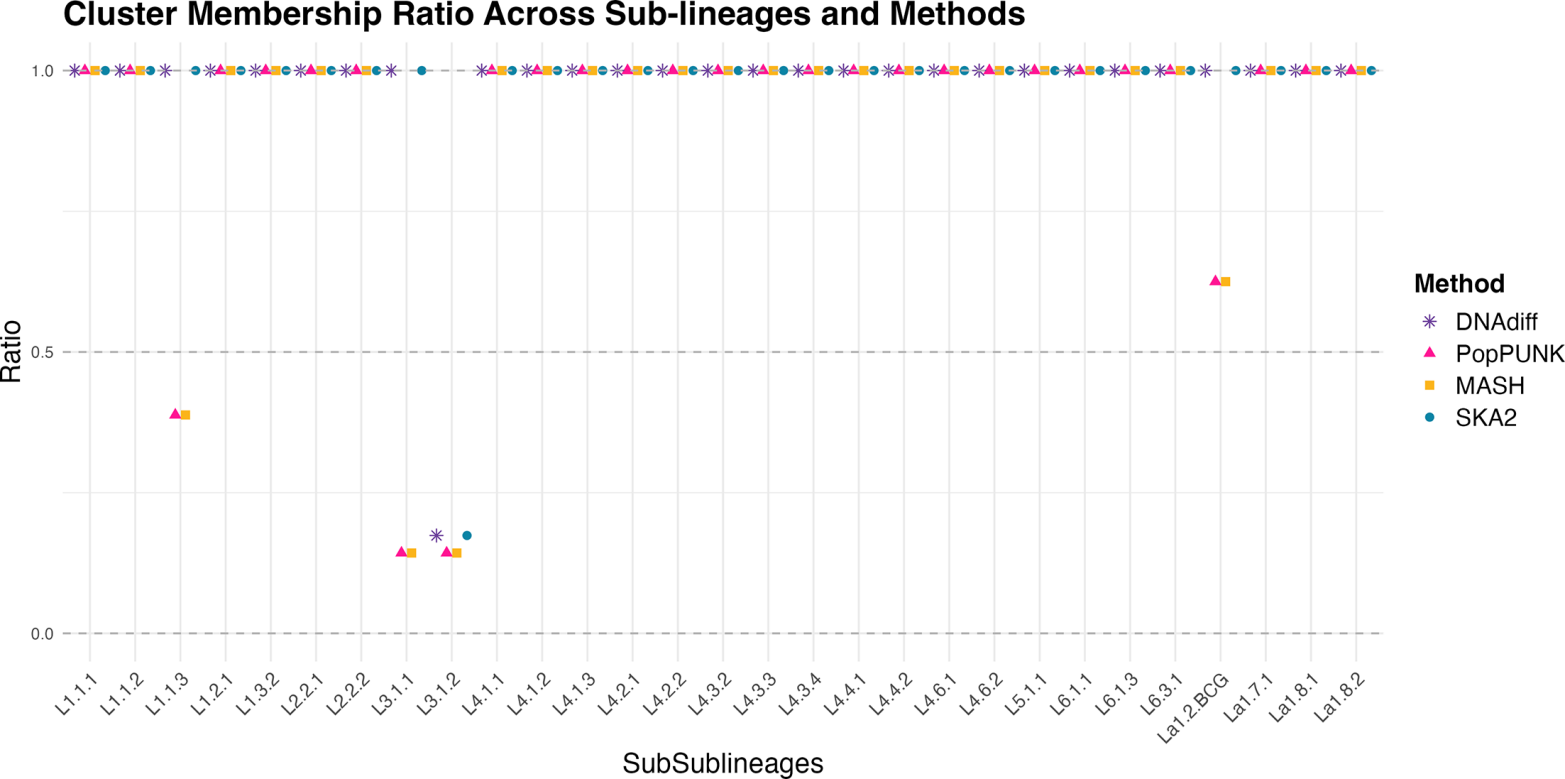
Sub-sub-lineage cluster membership accuracy ratio for each method using assembled simulated reads. A ratio of 1.0 indicates perfect agreement between each method’s clustering results and the TB-Profiler–assigned lineage groups. Ratios below 1.0 reflect over-clustering, where genomes from different sub-sub-lineage groups were incorrectly merged into the same cluster. A total of 410 genomes were included in this analysis.

**Fig. 5.**
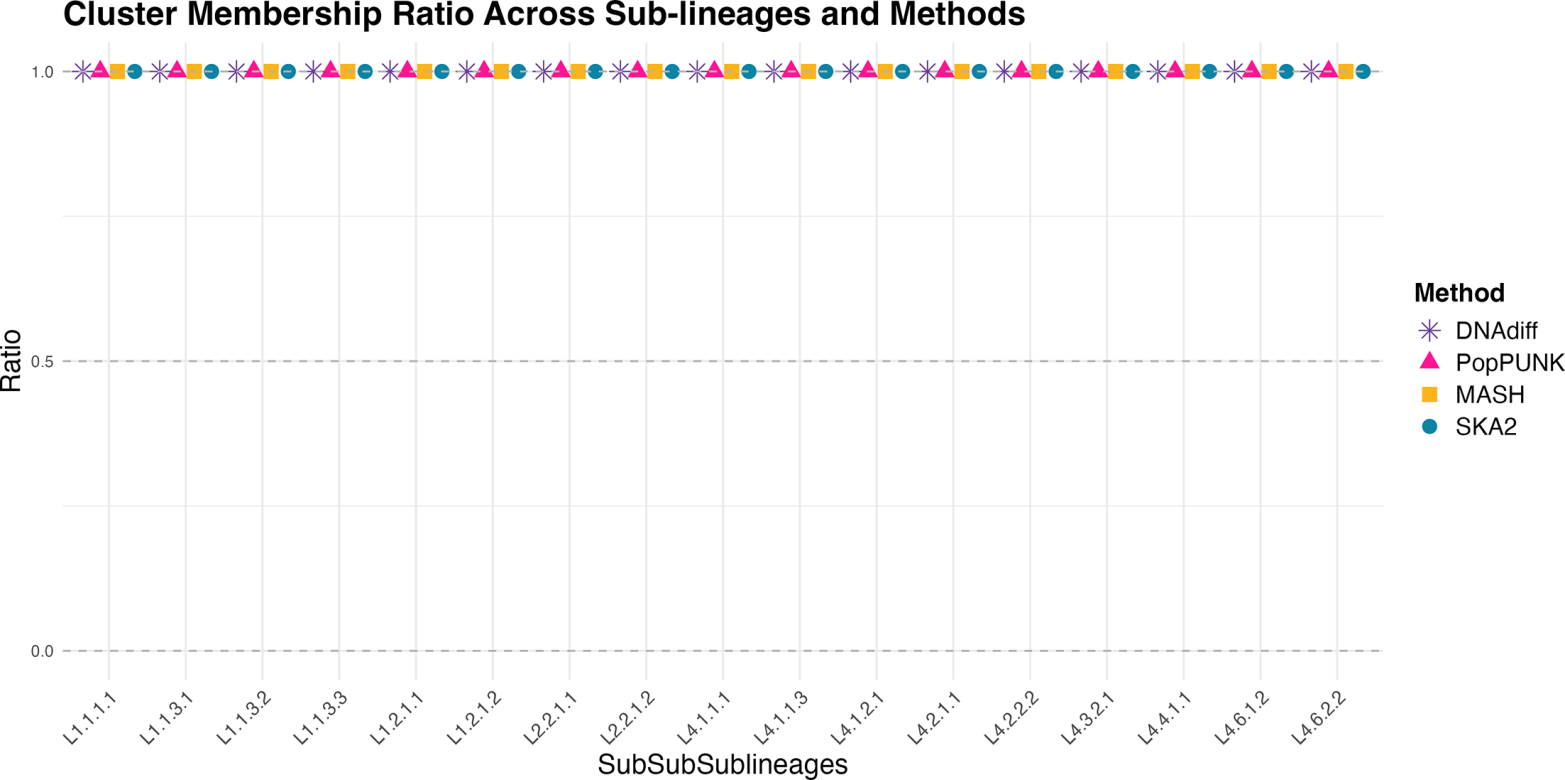
Sub-sub-sub-lineage cluster membership accuracy ratio for each method using assembled simulated reads. A ratio of 1.0 indicates perfect agreement between each method’s clustering results and the TB-Profiler–assigned lineage groups. Ratios below 1.0 reflect over-clustering, where genomes from different sub-sub-sub-lineage groups were incorrectly merged into the same cluster. A total of 157 genomes were included in this analysis.

#### Computational runtime and memory usage

Computational runtime and peak memory usage were measured for three reference-free methods (PopPUNK, MASH and SKA2), an alignment-based benchmark (DNAdiff) and a reference-based pipeline (MTBseq) using a dataset of 535 Mtb genomes. PopPUNK completed the analysis in ~2 min and MASH in ~5 min, with peak memory usage of around 1–1.2 GB RAM for both methods. SKA2 completed the analysis in ~40 min and reached a peak memory usage of ~10 GB RAM.

DNAdiff completed individual pairwise genome comparisons in seconds with a per-process memory usage of ~0.14 GB RAM, while processing the full dataset required ~10–12 days of serial compute time, or around 1 week of wall-clock time with moderate parallelization (8 CPUs). MTBseq required ~21 days to complete the analysis and reached peak memory usage of ~20 GB RAM per job. A summary of runtime and memory usage for all methods is provided in Table 1.

## Discussion

Genetic distance comparisons enable the evaluation of how effectively each method separates isolates within and between Mtb lineages. For the complete genomes, SKA2 produced the clearest separation between intra- and inter-lineage distances, closest to DNAdiff (Fig. 1a) and more concordant with TB-Profiler. In contrast, PopPUNK and MASH showed broader within-lineage distributions with noticeable overlap in the inter-lineage range, particularly for MASH. In the simulated read dataset, SKA2 maintained strong separation consistent with patterns derived from the reference-based read-mapping pipeline MTBseq (Fig. 1b). This agreement indicates that SKA2 produces lineage-level distance structure comparable to reference-based SNP calling when applied directly to reads. In contrast, PopPUNK and MASH displayed extensive overlap between intra- and inter-lineage distances (Fig. 1b), indicating reduced discriminatory power when applied directly to unassembled reads. After assembling the simulated reads, PopPUNK and MASH regained the expected lineage-level separation (Fig. 1c), more closely resembling the patterns observed for complete genomes, and SKA2 remained highly accurate and consistent across the different input datasets.

The relatively uniform distance distributions observed for PopPUNK and MASH when applied to simulated reads (Fig. 1b) reflect the underlying design assumptions of these *k*-mer-based methods. Both approaches estimate genetic distances using *k*-mer presence/absence and Jaccard-based similarity, which are primarily optimized for assembled genomes or long contiguous sequences [14,16]. When applied directly to short-read data, read fragmentation, uneven coverage and sequencing errors are expected to introduce noise into *k*-mer-based distance estimation [14,16], which can lead to compression of pairwise distances resulting in the observed uniform distance distributions.

Together, these findings highlight that SKA2’s split *k*-mer approach is more robust to diverse input data types, whereas PopPUNK and MASH are more sensitive to simulated read-level noise. Nevertheless, the residual overlap observed across all methods underscores the continued challenge of achieving high-resolution clustering using reference-free approaches, particularly in a genetically homogeneous pathogen like *M. tuberculosis*. This has important implications if such methods are to be implemented for recent transmission analyses, where the ability to resolve closely related isolates is critical for accurately inferring transmission events [28,29].

The distance-based trees generated by PopPUNK, MASH and SKA2 demonstrated that reference-free methods can effectively capture and delineate the broad population structure of *M. tuberculosis*, including human- and animal-adapted lineages. All three methods successfully reproduced the lineage groupings defined by TB-Profiler and those obtained through DNAdiff-based genome comparisons (Fig. S5). These findings support previous studies showing that reference-free *k*-mer-based approaches can approximate phylogenetic relationships in bacterial populations [14,30]. Despite their shared ability to resolve the major Mtb lineages, the resulting tree topologies revealed differences in branch lengths and resolution. Based on our results, SKA2 produced branching patterns more consistent with DNAdiff distances calculated from whole-genome alignments, recovering the major lineages as distinct clades and suggesting a higher resolution in capturing evolutionary relationships (Fig. S5).

The low resolution observed for PopPUNK and MASH in delineating several lineage groupings from simulated reads (Fig. S6) likely reflects the known limitations of applying *k*-mer-based methods with heuristic distance calculations directly to raw sequencing data. Raw reads can be more fragmented and often contain sequencing errors, which likely introduce false *k*-mers and reduce the accuracy of distance estimation [14,16]. Although both PopPUNK and MASH can run on unassembled reads, previous studies have reported that the best-hit strain is often incorrect due to noise in the raw data and that assembly or error-filtering significantly improves resolution [14,16]. After assembly with SKESA, both PopPUNK and MASH recovered the expected lineage grouping structure (Fig. S7), consistent with previous findings that assembly enhances the reliability of *k*-mer-based clustering analyses [14,16, 22].

We observed variation in how DNAdiff and the reference-free methods classified genomes into clusters corresponding to the lineage groups assigned by TB-Profiler when using complete genomes. Nevertheless, all methods showed consistent agreement with TB-Profiler-defined lineages at the broad lineage level (Fig. S8), highlighting both the reliability of reference-free approaches for broad lineage classification and the robustness of the SNP markers employed by TB-Profiler. The strong performance of these *k*-mer-based methods at this resolution highlights their practical relevance for public health surveillance, where rapid and accurate lineage identification is essential for informing outbreak investigations and guiding TB control strategies [6,31].

All methods showed a pattern of extended clustering when grouping isolates from sub-lineage L3.1 (Fig. S9), a group historically recognized for its poorly defined phylogenetic boundaries [32,33]. Notably, when using TB-Profiler as the classification baseline, several genomes assigned to L3.1 by DNAdiff and the reference-free tools were originally classified only as lineage 3, without sub-lineage designation. This pattern likely reflects the intrinsic complexity of defining sub-lineage L3.1, as lineage 3 is known to lack clear resolution at this level. For instance, a study from eastern Sudan also reported similar challenges, noting that although L3 was the predominant lineage, most isolates could not be confidently assigned to specific sub-lineages, highlighting current limitations in the sub-lineage classification of L3 [32]. Likewise, a study from the Kashgar Prefecture (China) reported the identification of four previously unclassified clades within lineage 3, suggesting that the current sub-lineage framework does not fully encompass the genetic diversity of L3 strains in the region [33].

These findings collectively highlight the need to refine current sub-lineage classifications to better reflect region-specific diversity within lineage 3. Furthermore, the observed extended clustering may suggest that these methods are capturing additional population structure not currently resolved by SNP-based classification schemes, potentially reclassifying some lineage 3 strains as L3.1. Future investigations could explore whether specific SNPs show complete differentiation between the groups defined by the reference-free methods (i.e. fixation index, F_ST_=1), providing a basis for improving and refining existing sub-lineage designations.

Beyond the challenges observed in L3.1, misclassification was identified within sub-lineage 4, where PopPUNK and MASH grouped genomes from sub-lineages L4.8, L4.9 and L4.7 into L4.6 (Fig. S9). This pattern suggests that these sub-lineages share a high degree of genetic similarity, potentially exceeding the resolving power of current sub-lineage definitions. The reduced discriminatory power when differentiating groups with minimal genomic divergence further highlights the broader challenge of achieving fine-scale resolution in *M. tuberculosis*, as previously described by [6]. Consistent with this observation, all these genomes displayed LAM spoligotype patterns, indicating a shared ancestral background that may obscure finer phylogenetic distinctions. Previous genomic studies described weak separation among several L4 sub-lineages and noted that many globally distributed lineage 4 groups exhibit overlapping population structures [34]. Additionally, Sabin *et al.* [35] reported that sub-lineages L4.7, L4.8 and L4.9 fall within the broader L4.10/PGG3 clade, a grouping originally defined by Stucki *et al*. [34], suggesting that their current subdivision may not accurately reflect true phylogenetic differentiation. Taken together, these findings imply that the grouping of these genomes by *k*-mer-based methods likely reflects genuine genomic similarity rather than methodological artefacts. Such results highlight that some apparent inconsistencies may arise from limitations in the existing taxonomic framework, emphasizing the need to re-evaluate sub-lineage boundaries within lineage 4 to ensure accurate representation of its evolutionary diversity. Notably, across all other evaluated sub-lineages, SKA2 correctly grouped genomes where lineage assignments were available, indicating high resolution applicable to a subset of the dataset.

A similar pattern of apparent misclassification was initially observed for some isolates previously designated as L1.1 and L1.2, which were grouped together by all methods, including DNAdiff. However, this discrepancy was later resolved following the updated redefinition of Mtb lineage 1 sub-lineages, where the former L1.2.2 was reclassified as L1.3 [36]. This revision aligned the clustering patterns observed across PopPUNK, MASH, SKA2 and DNAdiff with the revised lineage framework, confirming that the apparent inconsistency stemmed from outdated lineage definitions rather than methodological inaccuracies. This observation underscores the dynamic nature of Mtb taxonomy and highlights the importance of continuous refinement of reference databases and lineage nomenclature to ensure accurate genomic classification.

Interestingly, at the sub-sub-sub-lineage level, all methods achieved correct clustering concordance (Fig. S11), indicating strong resolution applicable to a specific subset of the dataset. However, it is important to note that only 151 genomes were assigned at this resolution, and most major Mtb lineages were not represented at this level. This limited diversity and reduced number of assignments may have simplified the clustering task, thereby inflating the apparent concordance. This may represent the practical limit of lineage subdivision, beyond which further resolution may reflect transmission clusters.

It is important to highlight that the comparatively reduced fine-scale clustering observed for PopPUNK in Mtb can be explained by the fact that the tool has been shown to recover biologically meaningful population structure across highly diverse bacterial pathogens [14]. In such organisms, pairwise genetic distance distributions effectively capture both within- and between-strain relationships, enabling stable and interpretable clustering [14]. In contrast, Mtb exhibits a strictly clonal population structure with low genome-wide genetic diversity, a defining feature of genetically monomorphic pathogens [37]. In this largely clonal context, genomic variation accumulates slowly, which can limit clustering resolution and compress genetic distance distributions.

Beyond clustering accuracy, practical considerations such as computational efficiency and ease of deployment are central to determining whether reference-free methods can be adopted in routine surveillance and clinical workflows. In this study, we compared the computational requirements of reference-free methods with alignment-based and reference-based workflows. Overall, reference-free approaches showed lower computational demands than DNAdiff and MTBseq when applied to the full dataset (Table 1). Although SKA2 requires greater memory usage than PopPUNK and MASH, it remains feasible in standard laboratory computing environments without access to high-performance infrastructure. In contrast, alignment-based and reference-based approaches were associated with substantially higher computational requirements when applied at scale (Table 1).

**Table 1.** Computational runtime and memory usage of evaluated methods using eight CPUs. Total runtime and peak memory usage measured for PopPUNK, MASH, SKA2, DNAdiff and MTBseq using a dataset of 535 Mtb genomes

	Runtime (full dataset)	Peak memory usage
PopPUNK	≈2 min	≈1–1.2 GB RAM
MASH	≈5 min	≈1–1.2 GB RAM
SKA2	≈40 min	≈10 GB RAM
DNAdiff	≈10–12 days (serial); ≈1 week with moderate parallelization	≈0.14 GB RAM per comparison
MTBseq	≈21 days	≈20 GB RAM per job

Together, these results demonstrate that reference-free methods substantially reduce computational barriers relative to alignment-based and reference-based workflows. While differences exist among reference-free tools in terms of runtime, memory consumption and resolution, all three approaches enable scalable genomic analysis without the extensive compute infrastructure typically required for read mapping or exhaustive alignment strategies. This supports the suitability of reference-free methods for routine strain typing and genomic epidemiology, particularly in resource-limited laboratory settings. However, differences in resolution capacity between tools should be carefully considered when selecting a method for downstream epidemiological or evolutionary analyses.

It is important to note that analyses based on full pairwise genetic distance calculations scale with the number of genomes analysed, such that larger datasets require increased computational resources.

In this study, we systematically evaluated the performance of three reference-free clustering methods, PopPUNK, MASH and SKA2, against the TB-Profiler-defined lineages, DNAdiff-derived lineage groupings and the reference-based MTBseq pipeline, to assess their applicability for strain typing and cluster definition.

Overall, reference-free methods applied to high-quality genome assemblies produced results comparable to current standard practice for lineage-level classification and broad population-structure analysis, while substantially reducing computational requirements. However, finer-scale resolution varied, reflecting both methodological and biological constraints. While these findings highlight the promise of reference-free approaches for rapid lineage-level classification and surveillance, some limitations should be acknowledged. The reliance on current SNP-based definitions, where some genomes lack sub-lineage designations, may limit resolution and overlook subtle or emerging diversity, underscoring the need for continued refinement of genomic markers and classification frameworks. Future work should expand evaluations to include geographically diverse collections, and routine sequence reads, beyond simulated data, to explore how reference-free approaches can support not only lineage delineation but also recent transmission analyses in real-world epidemiological contexts.

## Supplementary material

10.1099/mgen.0.001759 Supplementary Material 1.

10.1099/mgen.0.001759 Supplementary Material 2.

## Abbreviations

DNAdiff: genome comparison tool from the MUMmer package
MASH: MinHash-based sequence distance estimation
Mtb: *Mycobacterium tuberculosis*
NJ: neighbour-joining
PopPUNK: Population Partitioning Using Nucleotide *k-*mers
SKA2: split *k*-mer analysis
TB: tuberculosis
WGS: whole-genome sequencing
