# Supplementary material for: Reference-free clustering as an epidemiological tool for Mycobacterium tuberculosis lineage typing
Source: Microb Genom. 2026 Jun 24;12(6):001759. doi: 10.1099/mgen.0.001759 (PMC13292789; doi:10.1099/mgen.0.001759)
Supplement: Supplementary Material 2. [file mgen-12-01759-s002.pdf]

Supplementary Figures

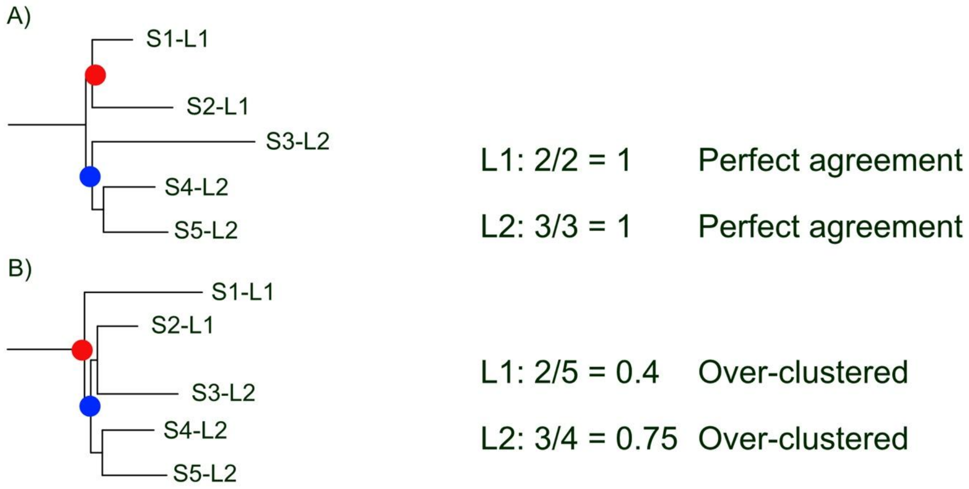

Figure S1. Illustration of lineage assignment agreement calculation. A lineage is assigned to each sample by TB-Profiler (indicated by the -L1 or -L2 in the sample name). A reference-free approach calculates distances between the samples and a dendrogram is created from that distance. The MRCA of each lineage (based on TB-profiler) is determined in the dendrogram (indicated by the red dot for L1 and blue dot for L2). The number of samples in that lineage is divided by the number descended from that MRCA to look for agreement. In A), the dendrogram separates that lineages properly, so perfect agreement is calculated, giving a score of 1. In B), the dendrogram incorrectly places one L1 sample in a clade with L2 samples, increasing the number of samples descended from each MRCA of each lineage, resulting in incorrect clustering.

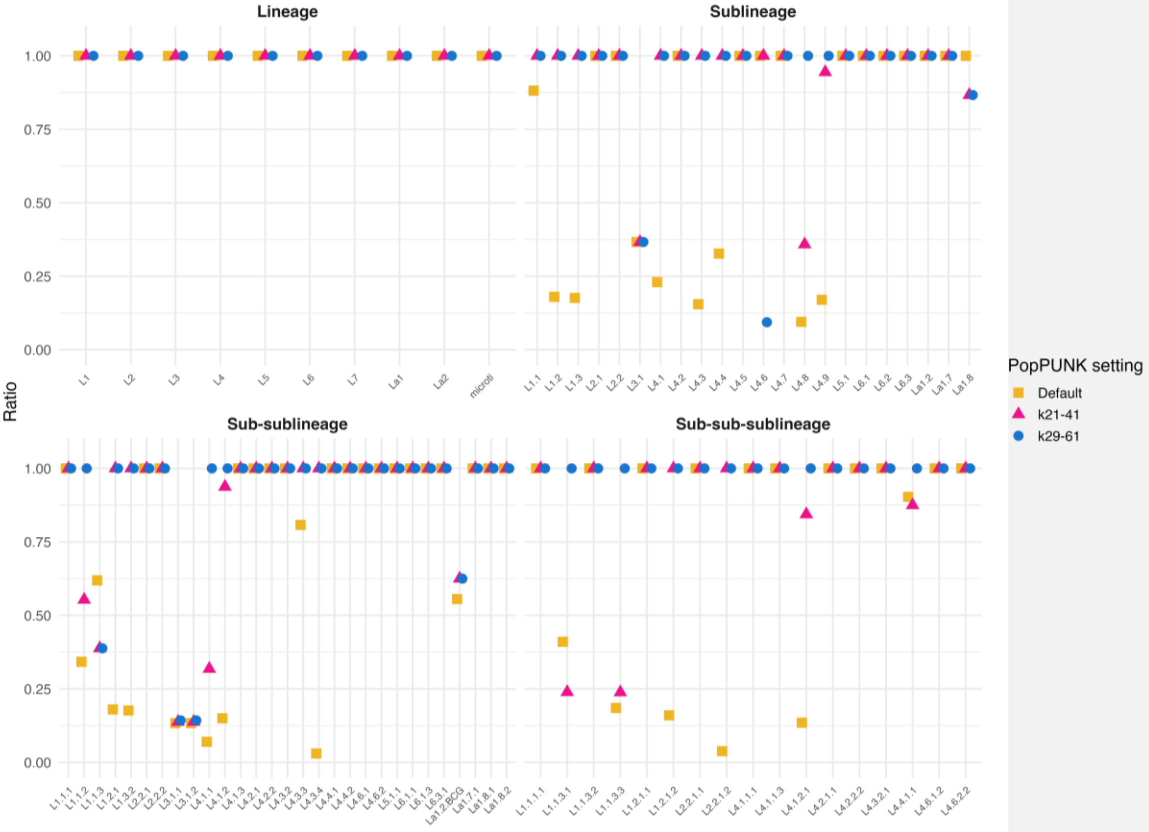

Figure S2. Effect of PopPUNK *k*-mer range on clustering resolution across lineage levels**.** Comparison of PopPUNK clustering results obtained using the default *k*-mer range (13–29), an intermediate range (21–41), and an extended range (29–61), evaluated at lineage, sub-lineage, sub-sub-lineage, and sub-sub-sub-lineage levels. Points show the agreement ratio for each lineage category under different *k*-mer parameterisations. The extended *k*-mer range (29–61) and sketch 100,000 was selected for downstream analyses. PopPUNK sketch size was increased from the default (10,000) to 100,000 for all analyses shown, as they provided higher resolution at finer population structure levels.

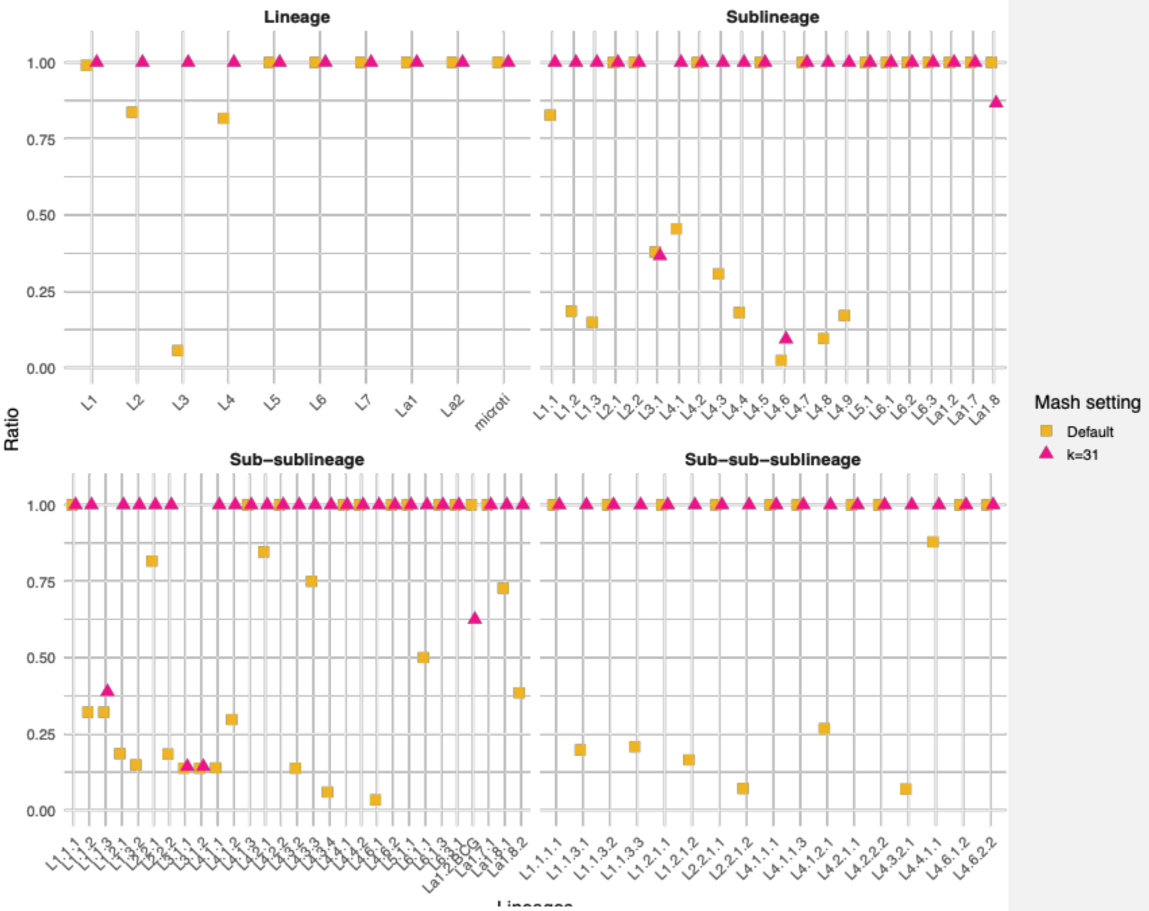

Figure S3. Effect of MASH *k*-mer and sketch size on clustering resolution across lineage levels. Comparison of Mash clustering results obtained using the default parameter settings ($k = 21$, sketch size = 1,000) and configurations using a larger *k*-mer size ($k = 31$) with increased sketch sizes up to 10,000, evaluated at lineage, sub-lineage, sub-sub-lineage, and sub-sub-sub-lineage levels. Points show the agreement ratio for each lineage category under different Mash parameterisations. A *k*-mer size of 31 and a sketch size of 10,000 were selected for downstream analyses, as they provided higher resolution at finer population structure levels.

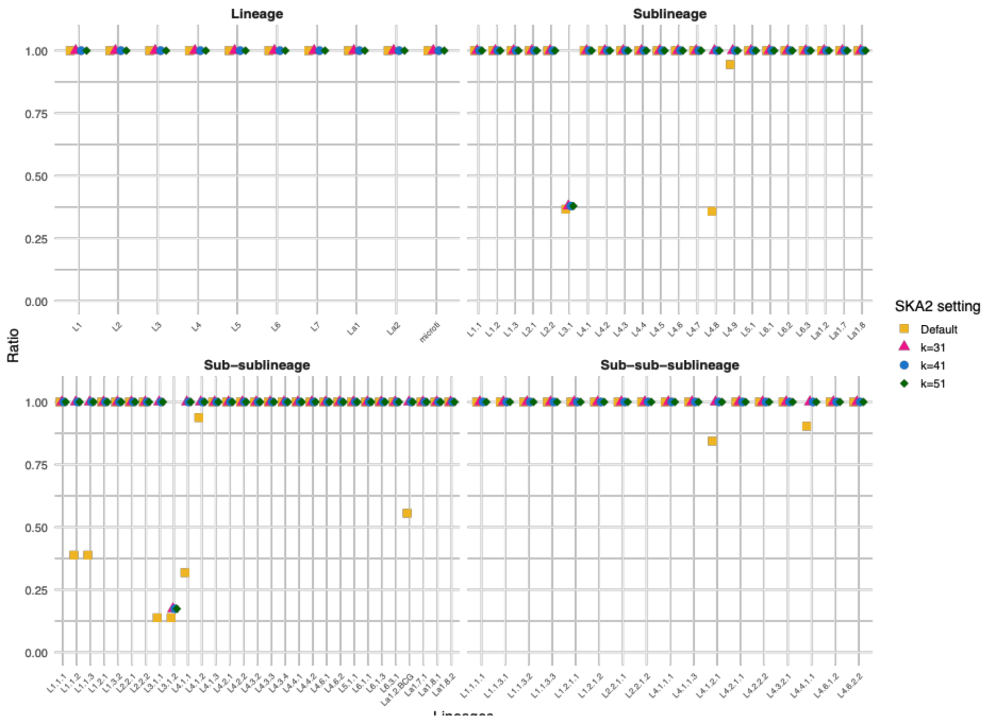

Figure S4. Effect of SKA2 *k*-mer size on clustering resolution across lineage levels. Comparison of SKA2 clustering results obtained using different split *k*-mer sizes (default, $k = 31$, $k = 41$, and $k = 51$), evaluated at lineage, sub-lineage, sub-sub-lineage, and sub-sub-sub-lineage levels. Points show the agreement ratio for each lineage category under different *k*-mer parameterisations. A split *k*-mer size of 31 was selected for downstream analyses, as it provided stable clustering across lineage levels without altering the overall population structure.

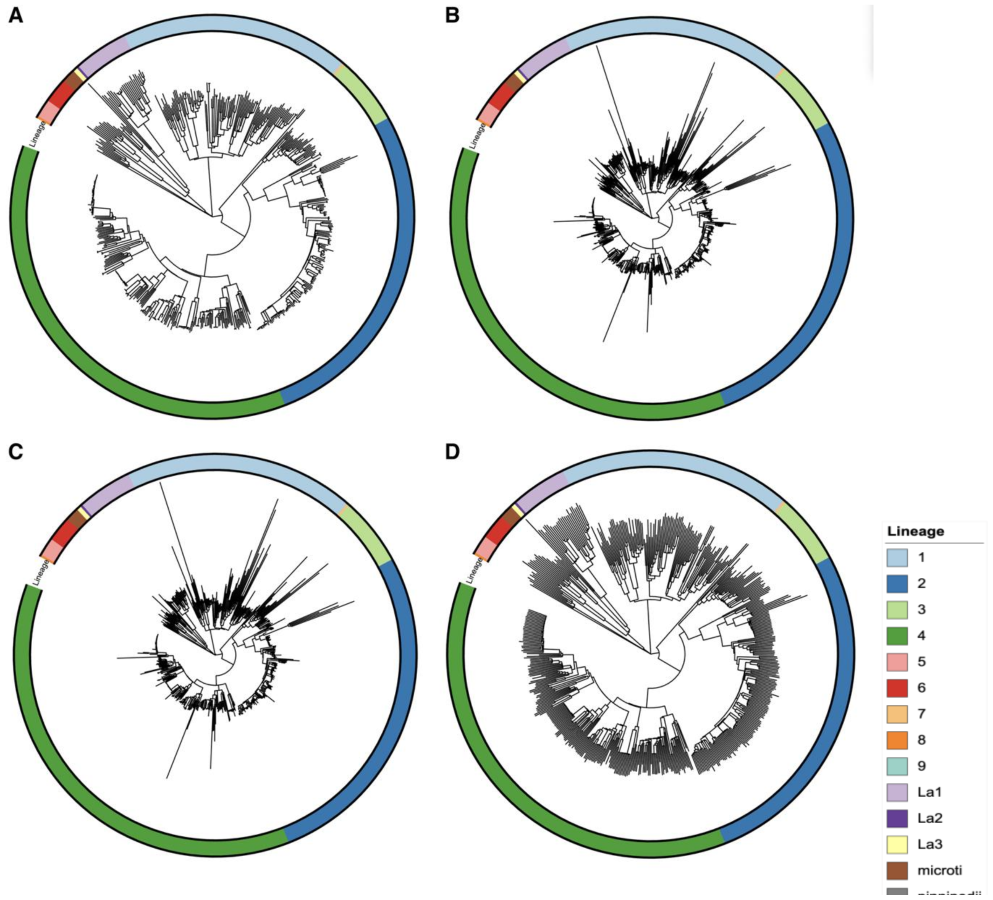

Figure S5. Delineation of lineages and relationships among the complete genomes. Distance-based NJ trees constructed from distance matrices generated by **DNAdiff (A)**, **PopPUNK (B)**, **MASH (C)**, and **SKA2 (D)**. All trees are rooted on the Lineage 8 strain**.**

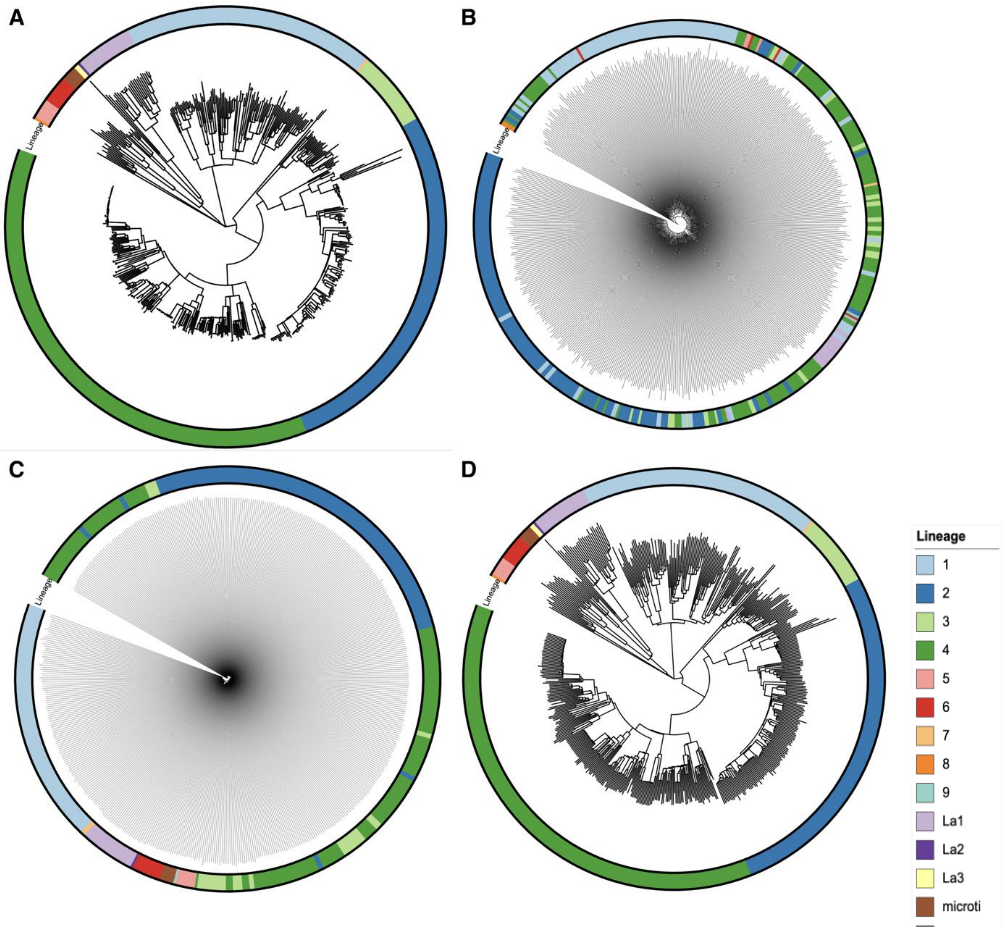

Figure S6. Delineation of lineages and relationships among the simulated reads. Distance-based NJ trees constructed from distance matrices generated by **MTBseq (A)**, **PopPUNK (B)**, **MASH (C)**, and **SKA2 (D)**. All trees are rooted on the Lineage 8 strain.

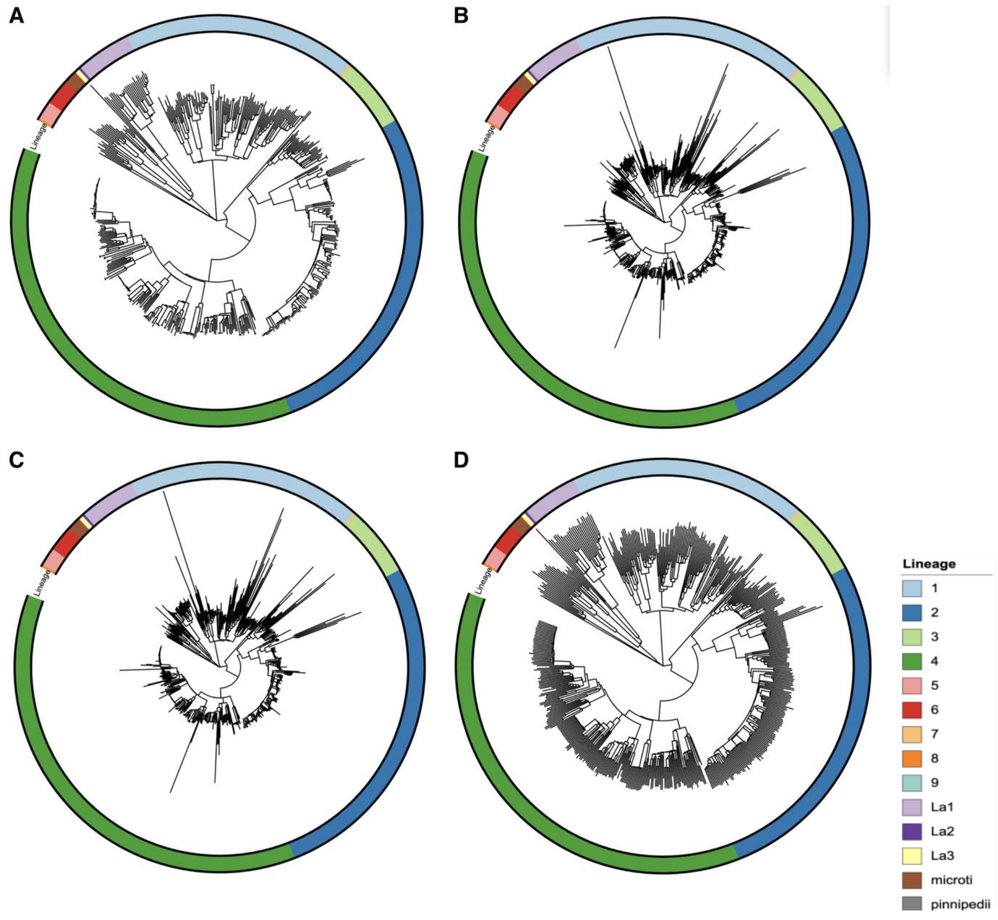

Figure S7. Delineation of lineages and relationships among the assembled simulated reads. Distance-based NJ trees constructed from distance matrices generated by **DNAdiff (A)**, **PopPUNK (B)**, **MASH (C)**, and **SKA2 (D)**. All trees are rooted on the **Lineage 8 strain**.

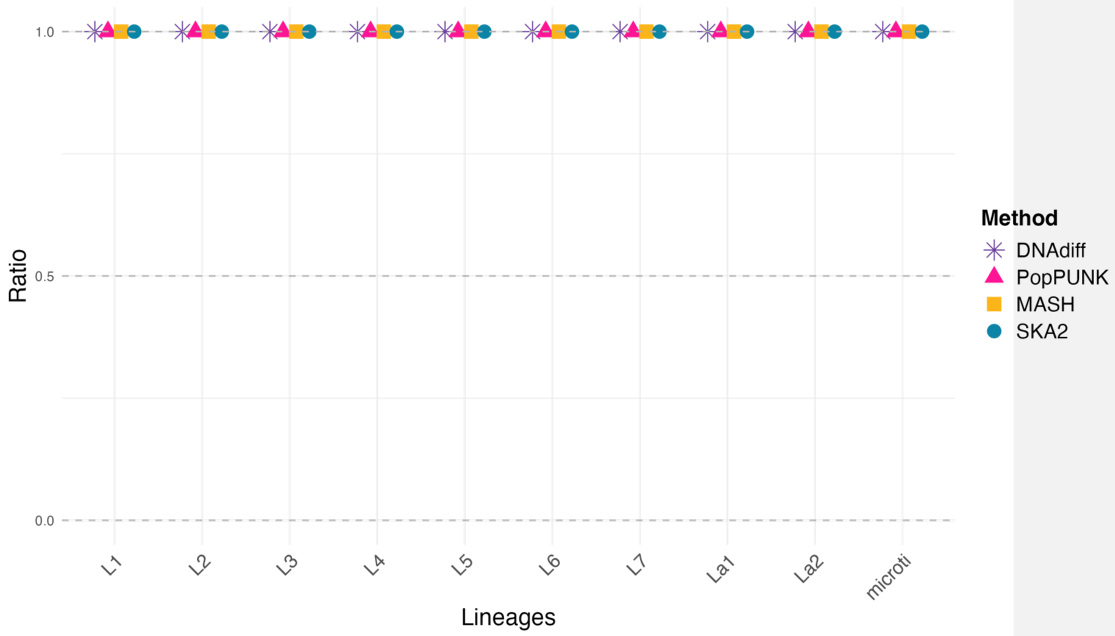

Figure S8. Lineage cluster membership accuracy ratio for each method using complete genomes. A ratio of 1.0 indicates perfect agreement between each method's clustering results and the TB-Profiler–assigned lineage groups. Ratios below 1.0 reflect over-clustering, where genomes from different lineage groups were incorrectly merged into the same cluster. A total of 535 genomes were included in this analysis.

Comment [DSMR1]: There are only for the analysis using compete genomes?

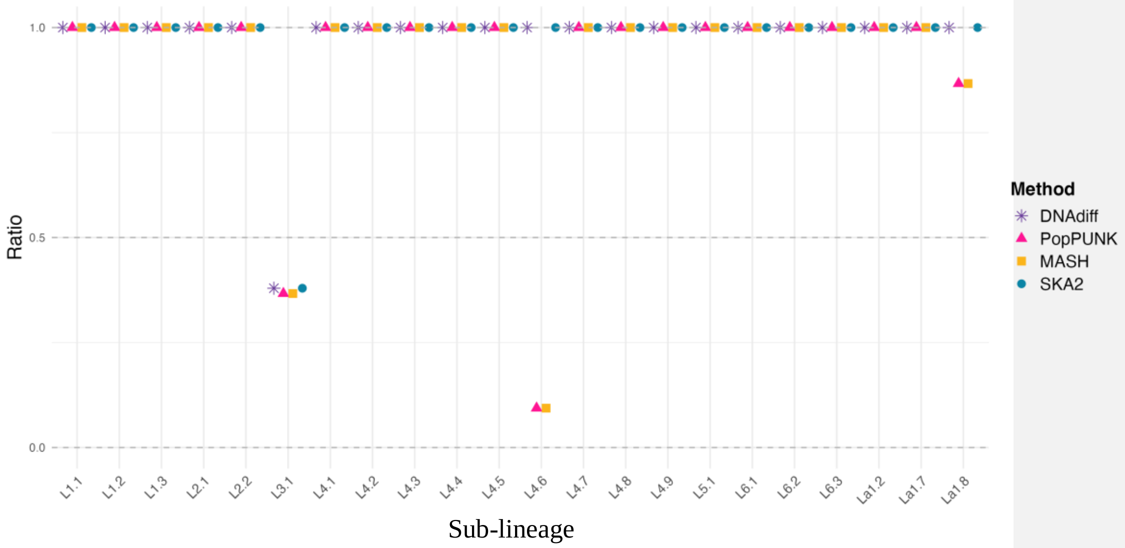

Figure S9. Lineage cluster membership accuracy ratio for each method using complete genomes. A ratio of 1.0 indicates perfect agreement between each method's clustering results and the TB-Profiler–assigned lineage groups. Ratios below 1.0 reflect over-clustering, where genomes from different lineage groups were incorrectly merged into the same cluster. A total of 494 genomes were included in this analysis.

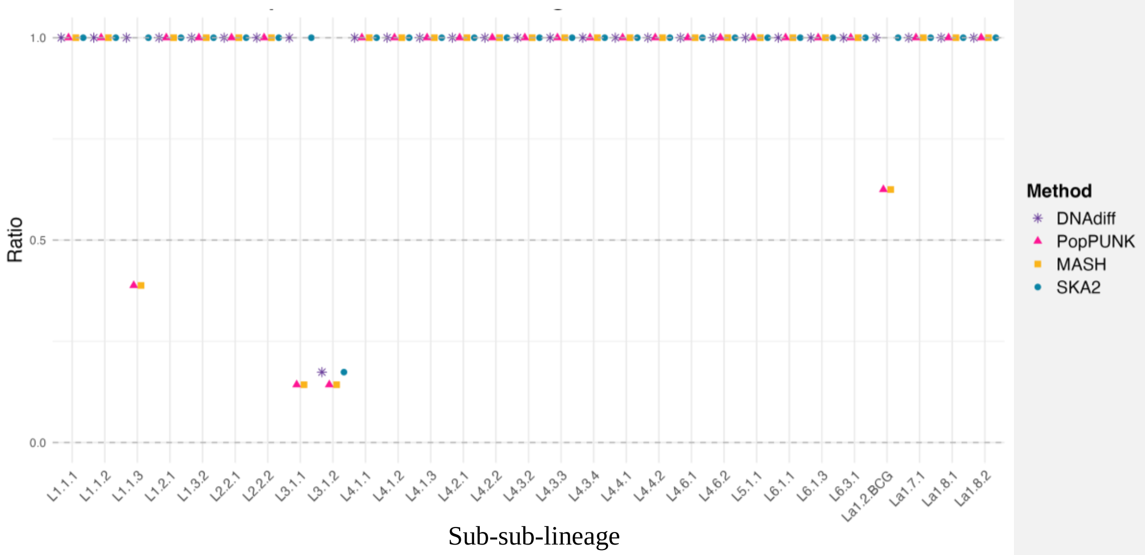

Figure S10. Lineage cluster membership accuracy ratio for each method using complete genomes. A ratio of 1.0 indicates perfect agreement between each method's clustering results and the TB-Profiler–assigned lineage groups. Ratios below 1.0 reflect over-clustering, where genomes from different lineage groups were incorrectly merged into the same cluster. A total of 410 genomes were included in this analysis.

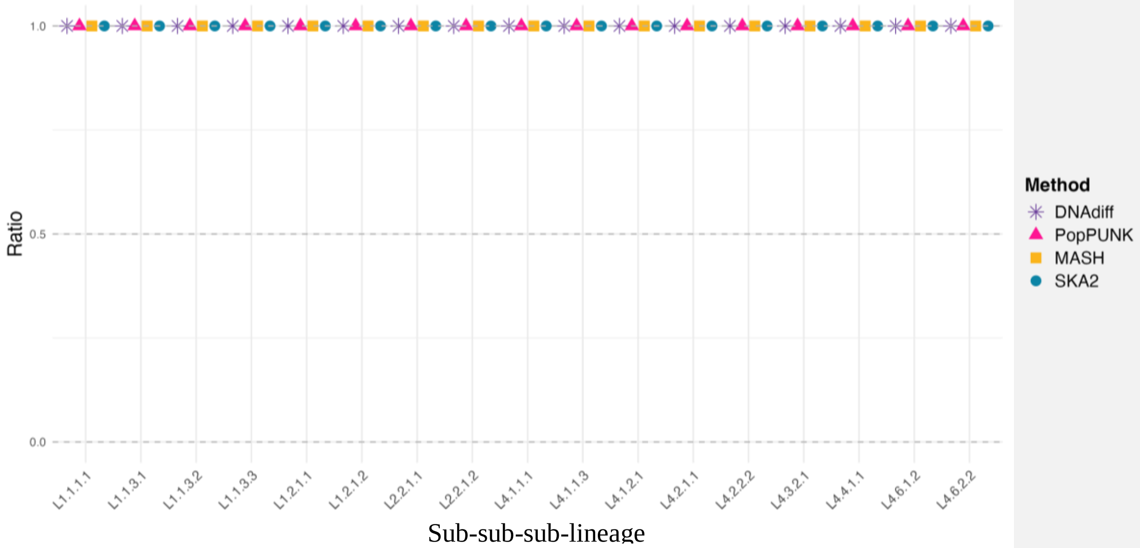

Figure S11. Lineage cluster membership accuracy ratio for each method using complete genomes. A ratio of 1.0 indicates perfect agreement between each method's clustering results and the TB-Profiler–assigned lineage groups. Ratios below 1.0 reflect over-clustering, where genomes from different lineage groups were incorrectly merged into the same cluster. A total of 157 genomes were included in this analysis.

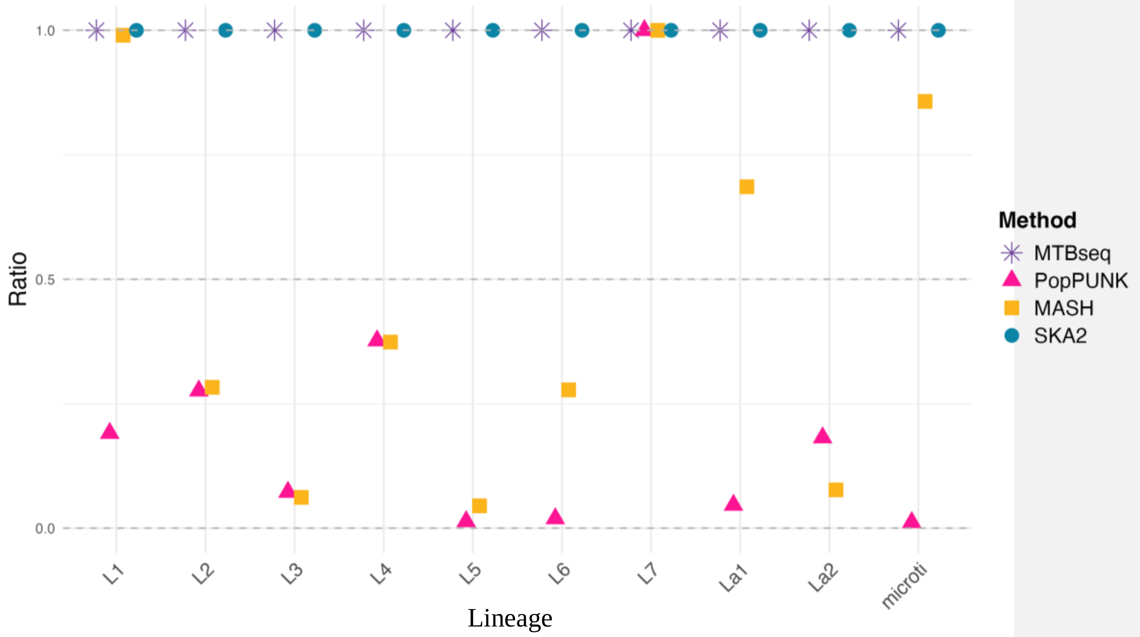

Figure S12. Lineage cluster membership accuracy ratio for each method using simulated reads. A ratio of 1.0 indicates perfect agreement between each method's clustering results and the TB-Profiler–assigned lineage groups. Ratios below 1.0 reflect over-clustering, where genomes from different lineage groups were incorrectly merged into the same cluster. A total of 535 genomes were included in this analysis.

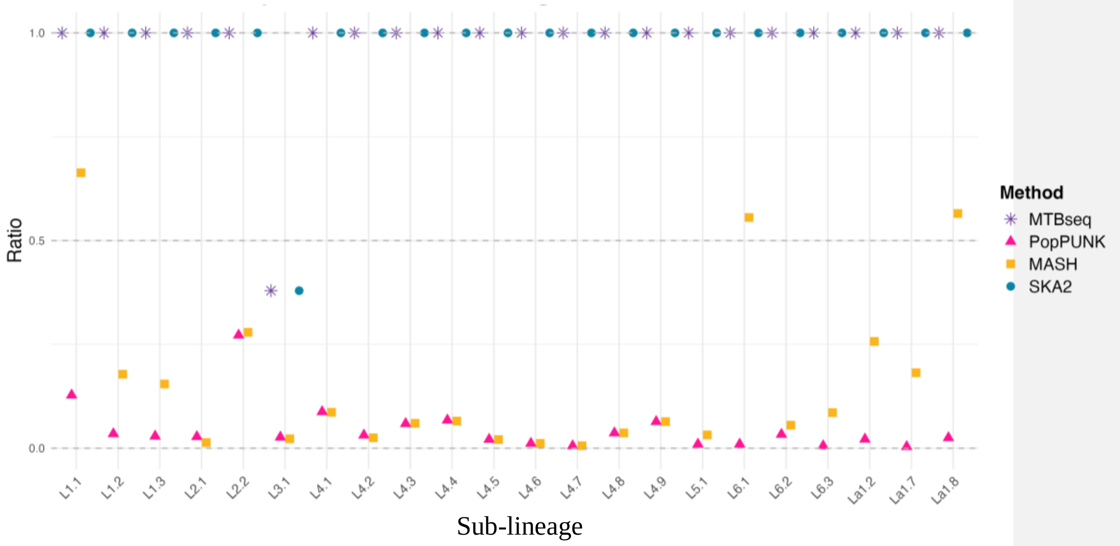

Figure S13. Lineage cluster membership accuracy ratio for each method using simulated reads. A ratio of 1.0 indicates perfect agreement between each method's clustering results and the TB-Profiler–assigned lineage groups. Ratios below 1.0 reflect over-clustering, where genomes from different lineage groups were incorrectly merged into the same cluster. A total of 494 genomes were included in this analysis.

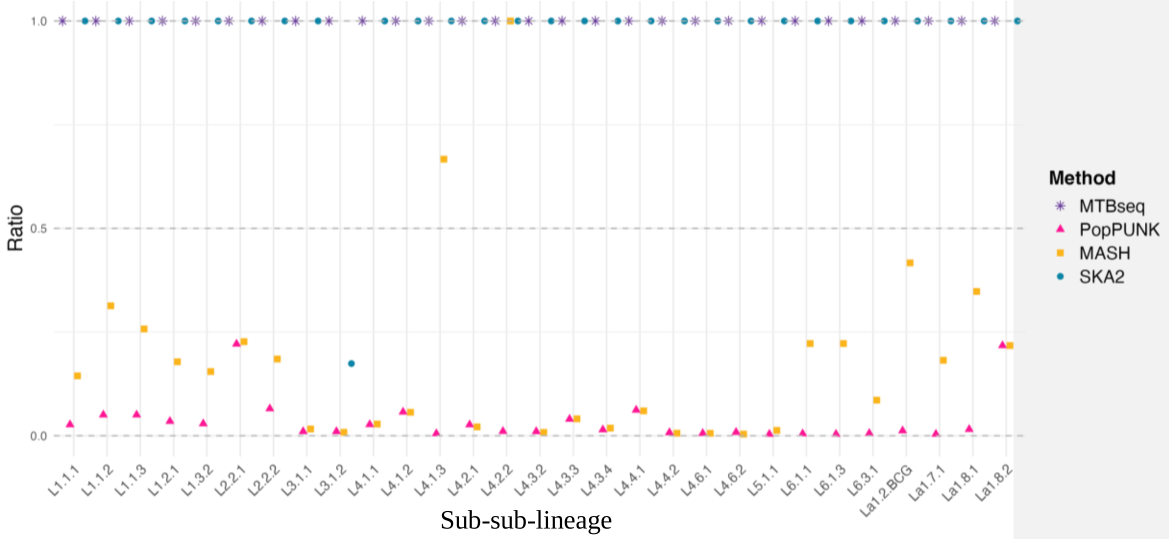

Figure S14. Lineage cluster membership accuracy ratio for each method using simulated reads. A ratio of 1.0 indicates perfect agreement between each method's clustering results and the TB-Profiler–assigned lineage groups. Ratios below 1.0 reflect over-clustering, where genomes from different lineage groups were incorrectly merged into the same cluster. A total of 410 genomes were included in this analysis.

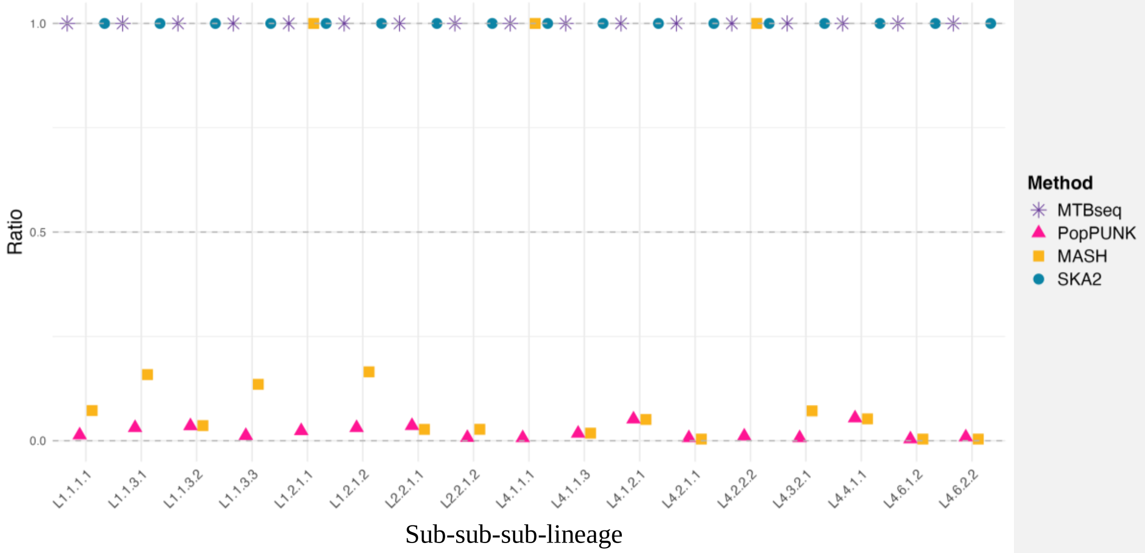

Figure S15. Lineage cluster membership accuracy ratio for each method using simulated reads. A ratio of 1.0 indicates perfect agreement between each method's clustering results and the TB-Profiler–assigned lineage

groups. Ratios below 1.0 reflect over-clustering, where genomes from different lineage groups were incorrectly merged into the same cluster. A total of 157 genomes were included in this analysis.
